# Aerial Insecticide Treatments for Management of Dectes Stem Borer, *Dectes texanus*, in Soybean

**DOI:** 10.1673/031.011.4901

**Published:** 2011-04-14

**Authors:** P. E. Sloderbeck, L.L. Buschman

**Affiliations:** Kansas State University, Southwest Research-Extension Center and Department of Entomology, 4500 E. Mary St., Garden City, KS. 67846.

**Keywords:** integrated pest management, sampling, soybean stem borer, yield losses

## Abstract

The Dectes stem borer, *Dectes texanus* LeConte (Coleoptera: Cerambycidae), is an increasingly important pest of soybean and sunflower in central North America. Nine large-scale field trials were conducted over a 3-year period to determine if Dectes stem borer could be managed with insecticide treatments. Aerial applications of lambda on July 6, 12 and 15 were successful in significantly reducing adults, but applications on July 1, 20 and 24 were less successful. These data suggest that for central Kansas two aerial applications may be required to control Dectes stem borers in soybean. Based on our experience the first application should be made at the peak of adult flight about July 5^th^ and the second application 10 days later. The local treatment schedule should be developed to follow the local Dectes stem borer adult emergence pattern. Treated aerial strips 59 m (195 ft) wide were not large enough to prevent reinfestation, but treated half-circles (24 ha or 60 acres) were successful in reducing in Dectes stem borer infestation of soybean. Sweep net samples of adults were not successful in identifying a treatment threshold, so treatment decisions will need to be based on field history of infestation. Further studies are needed to identify better sampling methods that can be used to establish treatment thresholds and to refine the best timing for treatments.

## Introduction

The Dectes stem borer, *Dectes texanus* LeConte (Coleoptera: Cerambycidae), is an increasingly important pest of soybean and sunflower in central North America. Producers are concerned because management options for this pest are limited (Lentz 1994; Sloderbeck et al. 2003; Buschman and Sloderbeck 2010; Sloderbeck et al. 2009). Older research suggested that crop rotation and stubble destruction would reduce damage from Dectes stem borers (Campbell and Van Duyn 1977; Rogers 1985), but such cultural practices appear to have lost their efficacy and in some cases are no longer compatible with current agronomic practices. Recently, Michaud et al. (2007b) suggested that sunflower could be used as a trap crop to reduce Dectes stem borer infestations in soybean.

Dectes stem borer larvae live inside the host plant, where they are protected from most foliar insecticide treatments. Eggs are inserted into the pith of newly expanded leaf petioles or into the tender stem, where larvae feed and tunnel in the pith inside the plant (Campbell 1980; Hatchett et al. 1975). The third instar larvae tunnel from the petiole into the stem, where they continue to feed and tunnel up and down the plant (Patrick 1973). As the plant approaches maturity, larvae move to the base of the plant and prepare overwintering chambers. They crawl up the stem to cut it off from the inside about 3 to 10 cm above ground (known as girdling). Girdled soybean stems break off and fall to the ground (i.e., lodge) and are difficult to retrieve with harvesting equipment; therefore, yields can be reduced. If the soybean crop is harvested promptly when plants first reach maturity and before girdling occurs, this lodging loss can be avoided. When harvest is delayed lodging increases and yield losses can be severe. In addition to lodging losses, physiological yield losses due to a loss of seed weight caused by the tunneling activity of Dectes stem borer can reach 10 to 15% (Campbell 1980; Buschman and Sloderbeck 2009; Daugherty and Jackson 1969). However, Michaud et al. (2007a) reported that there is no physiological yield reduction in sunflower infested with Dectes stem borer.

Dectes stem borer adult beetles emerge from the plant and it is the only stage that that would be exposed to insecticide treatments. In Kansas, the beetles emerge in late June and reach peak populations in early- to mid-July and then they decline through August. Females are present for ≈ 8 weeks and males for ≈ 4 weeks (Hatchett et al. 1975; Sloderbeck et al. 2003).

Although Dectes stem borer beetles appear to be susceptible to several insecticides (Cambell and Van Duyn 1977; Kaczmarek 2003), these chemicals have not generally been successful in reducing Dectes stem borer infestations (Campbell and Van Duyn 1977; Laster et al. 1981, Charlet et al. 2007a, 2007b). There are only a few reports of insecticide applications that did reduce the Dectes stem borer infestations. Seymour et al. (2000) reported that carbofuran (FMC Corp., Ag. Prod. Group, www.fmccrop.com/) reduced the Dectes stem borer infestation in sunflower. Knodel et al. (2009) reported that the experimental HGW86 10OD (DuPont Ag. Prod., www.dupont.com/Agriculture/en_US/) reduced the Dectes stem borer infestation in sunflower. J. Whitworth (personal communication) found a 14% yield increase in large plot soybean treated aerially with Hero^™^ insecticide targeting Dectes stem borer beetles (bifenthrin plus zetacypermethrin, FMC Corp.). Recently, it has been shown that some currently unlabeled systemic insecticides can be used to target larvae inside the plant and that these treatments can reduce Dectes stem borer infestations in soybean (Buschman et al. 2005, 2006; Davis et al 2008a, 2008b; Niide 2008).

The reason insecticide treatments have not been successful in controlling the Dectes stem borer appears to be because adults are present for such a long period during the summer and, therefore, are able to re-infest treated areas, particularly when they are small plots (typically 4 rows by 5 to 15 m). Therefore large-scale field trials were done to determine the feasibility of using large aerial plots and multiple insecticide applications to target the beetles in order to reduce Dectes stem borer infestations in soybean.

## Materials and Methods

**Figure 1.**
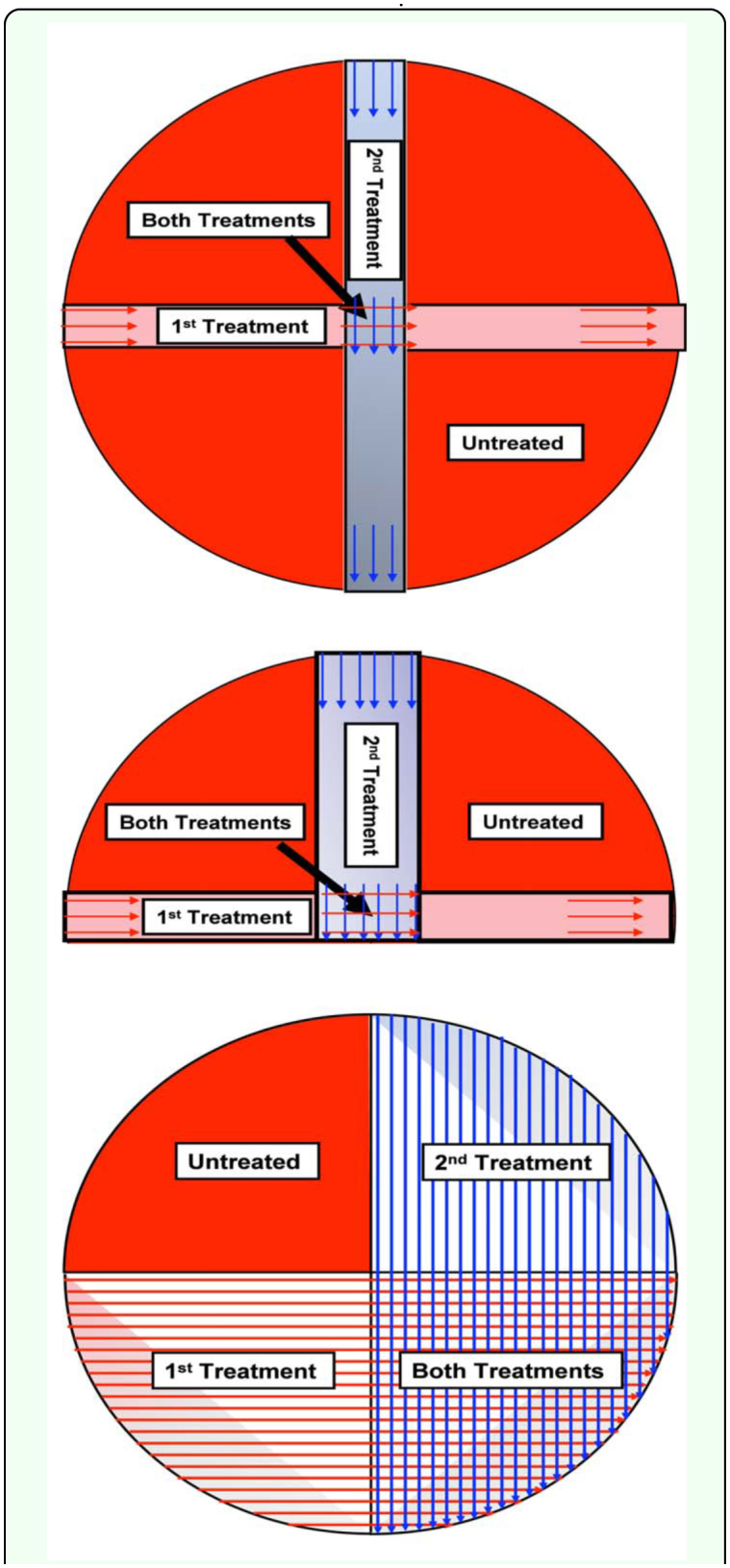
Arrangement of treatments in the large center-pivot circle fields (≈120 acres) for the aerial trials of 2001–2003. A. In 2001 ,the first treatments were made flying east-west across the center of the field, and the second treatments were applied flying north-south across the center of the field. The center received both treatments, and the bulk of the field outside the treated swaths remained untreated. B. In 2001, one field was a half circle of soybean. The first treatments were applied flying east-west across the center of the center pivot. The second treatments were applied flying north-south, but the area was wider. C. In 2002 and 2003, the first treatments were applied to the south half of the field. The second treatments were applied to the west half of the fields. High quality figures are available online.

Three irrigated circles of soybean with a history of heavy Dectes stem borer infestation (lodging observed the previous year) were identified in Pawnee and Edwards counties, Kansas, in each of 3 years (nine total fields). In 2001, the insecticide treatments were applied by plane to strips across the center of each field. Treated strips were three swaths wide (59 m or 195 ft). The first treatment was applied on 6 July flying east-west, and the second treatment was applied on 20 July flying north-south across the center of the field (Figure 1A). The two treatments were applied 90 degrees to each other, so there were four types of treatments in each field: areas treated the first time only (east-west), areas treated the second time only (northsouth), an area in the center that was treated both times, and four untreated pie-shaped areas that served as checks. One field was a half circle, so the second treatment was expanded to six passes wide (119 m or 390 ft) (Figure 1B). Because the 2001 trial was not as successful as desired, the size of the treated areas was increased from three swaths to a full half circle (24 ha or 60 acres in each 49 ha or 120 acre field). In 2002 and 2003, another six center-pivot-irrigated soybean fields were identified for these trials. The first application was made to the south half of each field flying east-west (six fields) (Figure 1C). The second application was made to the west half of five fields and to the east half of one field flying north-south. This resulted in four quarter fields (12 ha or 30 acres) in each field with four different treatments: one treated the first time only, one treated the second time only, one treated twice, and an untreated check. In 2002, treatments were delayed until beetle populations reached a minimal threshold (one per 100 sweeps), so the first application was made to two fields on July 12 and to the third field on July 17. The second treatments were applied on July 24. In 2003, treatments were applied on schedule (the first on July 1 and the second on July 15). The pyrethroid, Warrior^™^(lambda-cyhalothrin, Syngenta Crop Protection, Inc., www.syngenta.com) was applied by airplane at 0.028, 0.026 and 0.028 kg ai/ha (0.025, 0.023 and 0.025 lb ai/acre) in 28 1/ha (3 gal/acre) of water in 2001, 2002 and 2003, respectively.

Beetle populations were sampled before and after treatments using a sweep net. In 2001, the pretreatment samples were 100 sweeps at five locations (500 total sweeps) in each of the four treated areas (north, west, central and southwest) in the two full circles and four treatments (north, west, central and northeast) in the half field. The post treatment samples were 40 sweeps at five locations (200 total sweeps) in each treatment on 10, 16, 24 and 31 July (on 31 July the number of sweeps was reduced to 20 (100 total sweeps). Early in the season 100 sweeps were taken per location to detect beetle migration into the field. The sample number was reduced to 40 and then 20 sweeps per location to keep from overwhelming the sampler. In 2002 and 2003, samples were taken in each of the four quadrants using 20 sweeps at five locations (100 total sweeps). For analysis, all samples were converted to beetles per 100 total sweeps.

**Table 1.**
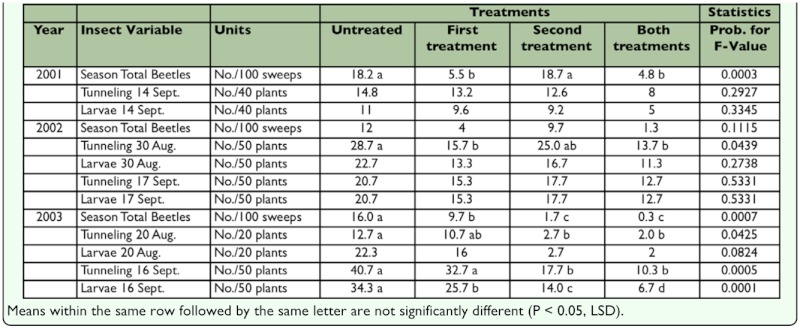
Dectes *texanus* observations in treated and untreated plots in 2001, 2002 and 2003.

Near the end of the season, plants were dissected to determine the number of plants with Dectes stem borer larvae and any tunneling. In 2001, a sample of 40 plants was dissected for each treatment on 14 September. In 2002, two samples of 50 plants were dissected for each treatment on 30 August and 17 September. In 2003, samples of 20 and 50 plants per treatment was made on 20 August and 16 September.

Data for each year were analyzed by 2-way ANOVA with four treatments and the three fields as replications. The means were separated by protected LSD (<0.05) MSTA Statistical Program (MSTAT Development Team 1988). Correlations between beetle counts and late season tunneling and larvae were done in Excel (Microsoft Office 2003).

## Results

**Figure 2.**
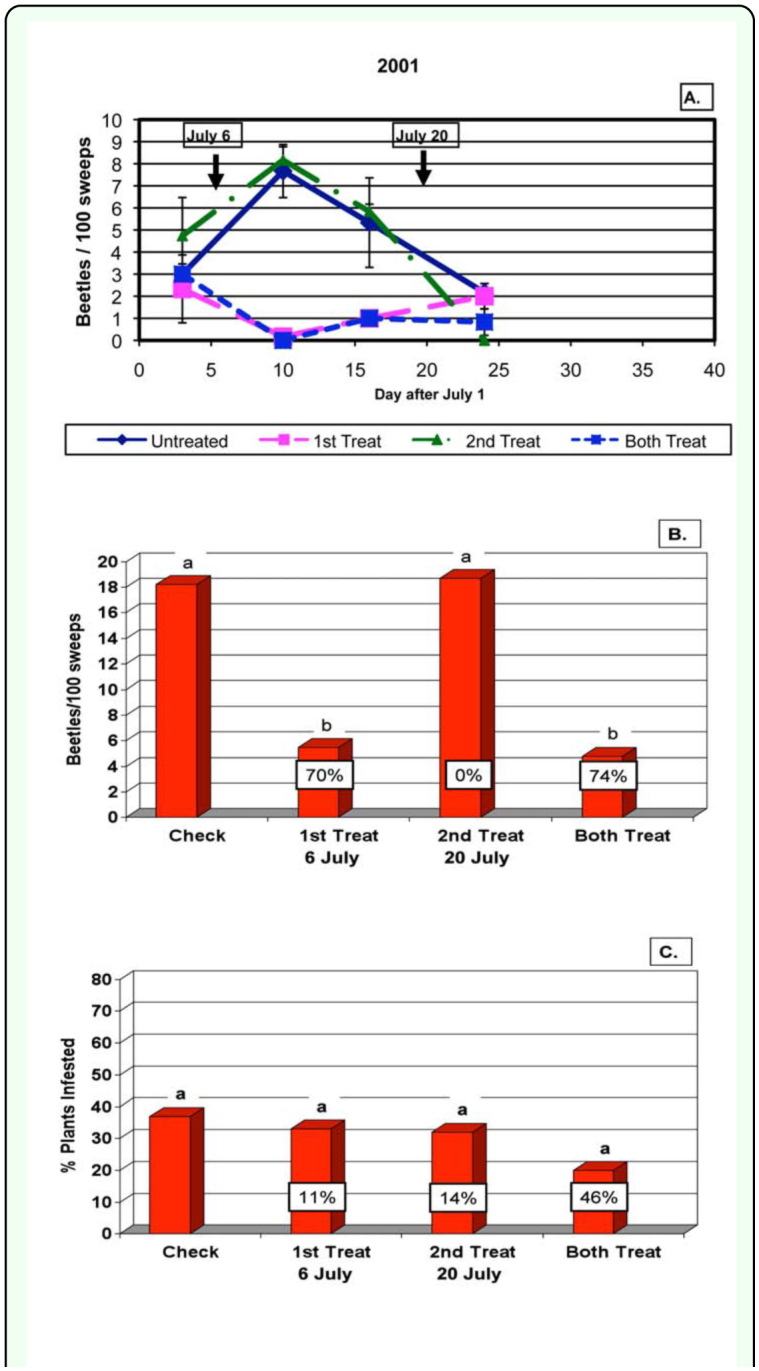
Results for 2001. A. *Dectes texanus* beetle numbers in 100 sweeps in the four treatments during the season. Arrows and dates indicate when insecticide treatments were made. B. Season-long total beetle numbers in the four treatments and percentage reduction relative to the control. C. Total percentage of plants tunneled in the four treatments and percentage reduction relative to the control. High quality figures are available online.

In 2001, average pretreatment beetle counts in the four areas of the field ranged from 0.8 to 3.0, 0.6 to 7.6 and 3.0 to 7.0 beetles per 100 sweeps in the first, second and third fields, respectively. The first treatment on 6 July was successful in reducing season-long beetle populations by 70%; the second treatment 14 days (20 July) later did not reduce populations, but the two treatments together reduced numbers by 74% (Figures 2A and 2B). Plants were dissected on September 14, and 35% of the plants were tunneled and 28% had larvae. Although treatments significantly reduced beetle populations, the percentage of plants with tunneling was only reduced 11% for the first treatment, 14% for the second treatment and 46% for the two treatments together (Table 1 and Figure 2C). This indicated the plot area was not large enough to keep beetles from reinfesting. Therefore, the experiment was repeated using larger plots the following year.

**Figure 3.**
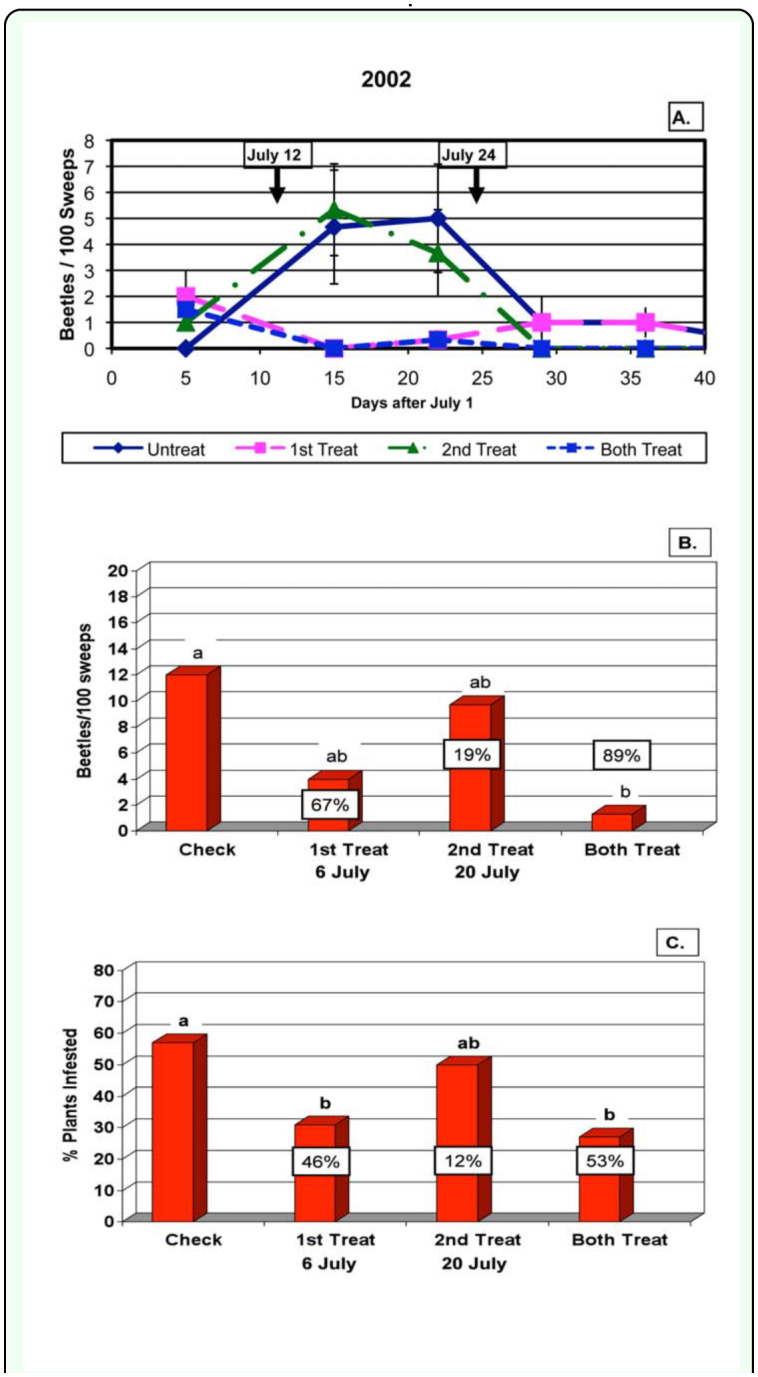
Results for 2002. A. Dectes *texanus* beetle numbers in 100 sweeps in the four treatments during the season, Arrows and dates indicate when insecticide treatments were made. B. Season-long beetle numbers in the four treatments and the percentage reduction relative to the control. C. Total percentage of plants tunneled in the four treatments and percentage reduction relative to the control. High quality figures are available online.

In 2002, average pretreatment beetle counts in the four areas of the field ranged from 0.0 to 3.0, 0.0 to 2.0 and 0.0 to 2.0 beetles per 100 sweeps in the first, second and third fields, respectively. Because beetle counts were low, the first treatments were delayed and not applied until 12 July (17 July for one field). This first treatment was successful in reducing season-long beetle populations in the treated areas by 67%, but the second treatment 12 days later on 24 July only reduced them by 19%, and the two treatments together reduced them by 89% (Figure 3A and 3B). A total of 50 plants were dissected on 30 August and again on 17 September. The infestation rate averaged 57 and 41% of plants with tunneling in the check (Table 1). The first treatment on July 12 significantly reduced the percentage of plants infested by 46%, the second treatments reduced them 12% and the two treatments together reduced them by 53% (Table 1 and Figure 3C).

**Figure 4.**
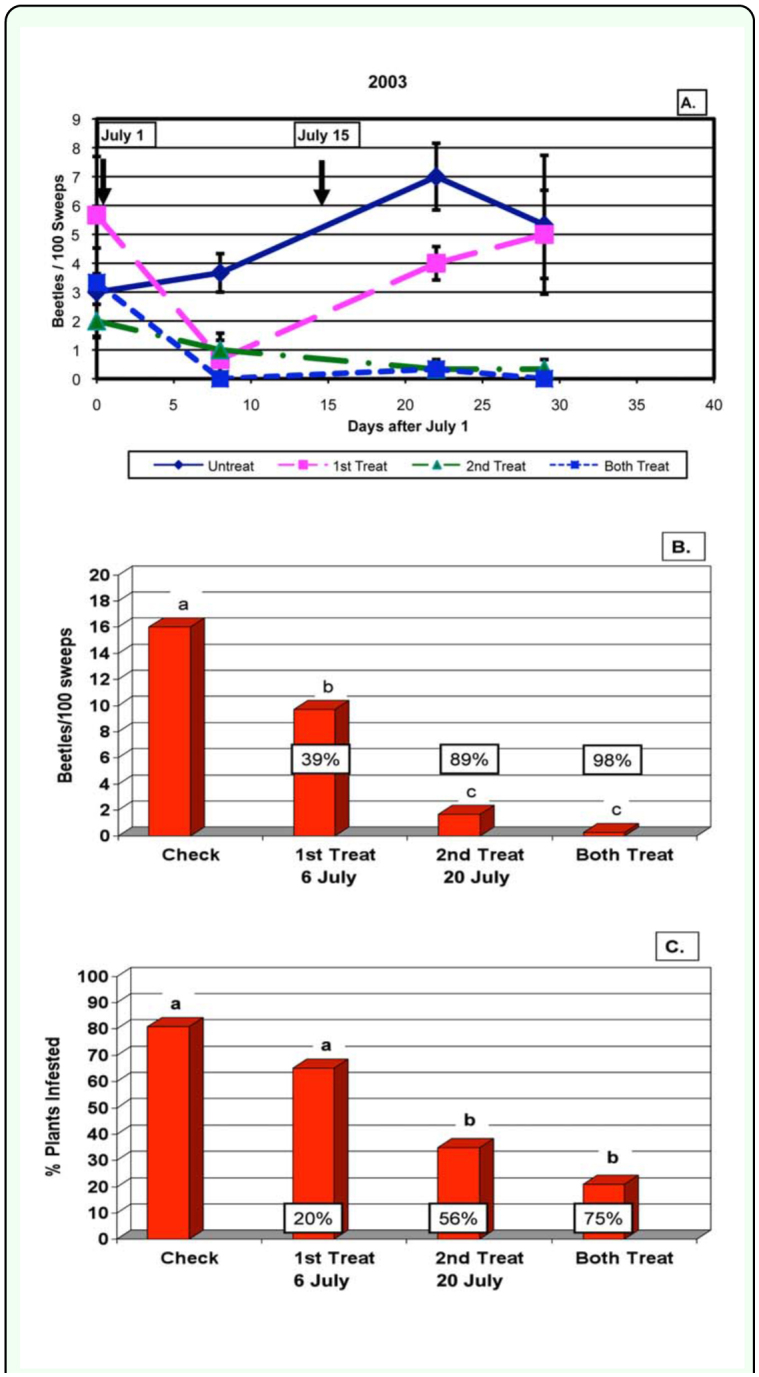
Results for 2003. A. Dectes *texanus* beetle numbers in 100 sweeps in the four treatments during the season. Arrows and dates indicate when insecticide treatments were made. B. Season-long beetle numbers in the four treatments and the percentage reduction relative to the control. C. Total percentage of plants tunneled in the four treatments and percentage reduction relative to the control. High quality figures are available online.

In 2003, average pretreatment beetle counts in the four areas of the field ranged from 0 to 3, 1 to 6 and 3 to 9 beetles per 100 sweeps in the first, second and third fields, respectively. Treatments were made on schedule on July 1 and July 15. The first treatment reduced season-long beetle populations by 39%, the second treatment reduced them by 89% and the two treatments together reduced them by 98% (Figure 4A and 4B). A total of 20 and 50 plants were dissected on 20 August and again on 16 September. The infestation rate averaged 82% of plants infested in the check plots. The first treatment reduced the percentage of infested plants 20%, the second treatment reduced them 56% and the two treatments together reduced them by 75% (Table 1 and Figure 4C).

Some insecticide applications were more effective than others in reducing season-long beetle populations. The treatments on July 1 and 6 were somewhat early and gave 39 and 70% control of beetles, but they gave only 16 and 11% control of infested plants. The treatments on July 12 and 15 were more effective and gave 67 and 89% control of beetles and 46 and 78% control of infested plants. The applications on July 20 and 24 were too late and gave only 9 and 19% control of beetles and 14 and 12% control of infested plants. Therefore, in Pawnee and Edwards counties, the ideal first treatment date would be between July 1 and 6, and the second treatment should be made approximately 10 days later, between July 15 and 17. Although single treatments gave significant control (up to 78%), two treatments gave better control (53 and 89%). In 2 years, the first treatments gave better control than the second treatment, but in the third year, the second treatment gave better control than the first treatment. Until further research is conducted to determine the ideal timing of insecticide treatments, two treatments are recommended to provide good control.

## Discussion

Data from this study did not support a treatment threshold. When the pre-treatment beetle counts averaged over all plots were correlated with end of season infestation in the check plots the correlation coefficients were only 0.023 (tunneled plants) and 0.023 (larvae), respectively (n=8). When pretreatment beetle counts in each plot were correlated with end of season infestation in each plot the correlation coefficients were 0.081 (tunneled plants) and 0.070 (larvae), respectively (n=32). When season total beetle counts for all plots were correlated with end of season infestation in each plot the correlation coefficients were 0.138 (tunneled plants) and 0.146 (larvae), respectively (n=32). In 2001, total beetle counts per 100 sweeps averaged 18.2, but the percentage of plants infested at the end of the season was only 37%. In 2002 and 2003, total beetle counts per 100 sweeps averaged only 12 and 16, but the percentage of plants infested at the end of the season averaged 57 and 64%, respectively. Many plots had zero or only one beetle per 100 sweeps in the pretreatment sample, but the plots were heavily infested at the end of the season. Since beetle counts were not correlated with end of the season infestations they cannot be relied on to trigger insecticide treatments.

Good Integrated Insect Pest Management practice would be to apply insecticide treatments only when the pest populations reach a treatment threshold. However, since there is virtually no correlation between beetle counts and end of season infestations, sweep net counts cannot be used to establish a treatment threshold. We therefore suggest that treatment decisions be based on the history of Dectes stem borer infestation in the field (or adjacent fields). Fields with >50% of plants infested the previous year will likely be heavily infested next year. Fields in the vicinity of the heavily infested field that are planted to soybean the next year should be considered vulnerable to heavy infestation by the Dectes stem borer and may need to be treated to control this pest. We do not support the treatment threshold of one beetle per 10 sweeps proposed by FMC Corporation (2009), because the correlation between beetle counts and end of the season infestation is so low.

On the other hand sweep net samples that are made on a regular schedule do seem to document the seasonal occurrence of beetle populations. It may be possible to use sweep net samples to predict the annual trends in Dectes stem borer populations to help time insecticide applications to peak pest populations. Such counts should be used retrospectively determine how well treatment applications were timed relative to the overall population trend. Based on our experience the first application should be made at the peak of adult flight and the second application 10 to 14 days later. Timing of insecticide treatments for other regions will be slightly different depending on the temperature regimes for these regions. Adult emergence occurs earlier in the south (Texas Panhandle) and later in the west and north. The local treatment schedule should be developed to follow the local adult emergence pattern. These trials provide some guidance on how to start developing a local treatment schedule.

It should be noted that there are few other insect pests of soybean when these insecticide treatments are applied (central and western Kansas in June and July). Bean leaf beetles, *Ceratoma trifurcate*, soybean aphids, *Aphis glycines*, stink bugs and various defoliating pests are generally more significant farther east and their occurrence is later in the season (except for bean leaf beetle which is earlier).

This study showed that properly timed aerial insecticide applications can be used to manage Dectes stem borer, but the timing, economics and treatment thresholds for such applications need to be more fully studied. At this time two insecticides appear to be available for use, Warrior^™^ (lambda-cyhalothrin, Syngenta Corp.) which is labeled for use in soybean, but it is not specifically labeled for the Dectes stem borer, and Hero^™^ (bifenthrin plus zetacypermethrin, FMC Corp.) which is labeled for Dectes stem borer in soybean.
